# Reducing the rate of cesarean delivery on maternal request through institutional and policy interventions in Wenzhou, China

**DOI:** 10.1371/journal.pone.0186304

**Published:** 2017-11-20

**Authors:** Yushan Yu, Xiangyang Zhang, Caixia Sun, Huijie Zhou, Qi Zhang, Chun Chen

**Affiliations:** 1 School of Public Health and Management, Wenzhou Medical University, Wenzhou, China; 2 Department of Preventive Medicine, Medical School of Shihezi University, Shihezi, China; 3 First Affiliated Hospital of Wenzhou Medical University, Wenzhou, China; 4 Wenzhou People’s Hospital, Wenzhou, China; 5 School of Community and Environmental Health, College of Health Sciences, Old Dominion University, Norfolk, VA, United States of America; Helsingin Yliopisto, FINLAND

## Abstract

The objective of this study was to evaluate the effect of institutional and policy interventions on reducing the rate of cesarean delivery on maternal request (CDMR) in Wenzhou, China. Institutional interventions included health education, painless delivery introduction, and doula care. Additionally, a series of health policies were developed by the Chinese central and local governments to control cesarean section rates, mostly through controlling CDMR rates. We conducted a pre-/post-intervention study using 131,312 deliveries between 2006 and 2014 in three tertiary-level public hospitals in Wenzhou, China. Chi-square tests and predictive models were used to examine changes in the CDMR rate before and after institutional and policy interventions. After institutional interventions were introduced, the overall CDMR rate increased from 15.76% to 16.34% (p = 0.053), but the average annual growth rate (AAGR) of the overall CDMR rate quickly declined from 20.11% to -4.30%. After policy interventions were introduced, the overall CDMR rate, the AAGR of the overall CDMR rate, and the probability of performing CDMR declined. Further, the overall probability of a woman undergoing CDMR decreased in all three age groups (group one: <24; group two: 24–34; group three: >34) after institutional and policy interventions. These results show that institutional and policy interventions can reduce the CDMR rate. Additionally, the CDMR rate should be included in hospitals’ performance assessment matrix to reduce the CDMR rate further.

## Introduction

Cesarean section (CS) rates have increased significantly in recent years. The CS rate was 13.1% in 2000 and 16.9% in 2012 in developing countries, including China [1]. Medically necessary CS is essential to protect mothers’ and newborns’ lives, but unnecessary CS, cesarean delivery on maternal request (CDMR), should be reduced [2]. In mainland China, the CS rate was 54.9% in 2011 [3], more than triple the ideal rate of 15% recommended by the World Health Organization (WHO) [4]. More notably, the CDMR rate, the main indication for unnecessary CS, was 28.43% in 2011 [3]. Additionally, CDMR increases numerous risks, such as a higher probability of maternal infection [5], increased neonatal respiratory morbidity [6], higher risk of maternal death [7], and side effects in subsequent pregnancies [8]. Decreasing the CDMR rate was a crucial and effective way to control the unnecessary CS rate in China. China’s universal two-child policy took effect in 2016, which allows couples to have two children [9]. Moms with their second pregnancies are interested to deliver the second child via CS and the number of CDMRs is likely to increase. Therefore, reducing the high CDMR rate should be a high priority on the policy agenda to protect maternal and child health.

Since 2000, a few Chinese hospitals have started to control the unusually high CS rates with health education [10, 11], painless delivery [12], doula delivery [13], and psychological comforting and training programs for midwives and obstetricians [14]. After 2011, the Chinese central and local governments successively developed specific policies to control the CS rate through reducing the CDMR rate [15–18]. Remarkably, the CS rate was the main monitoring indicator for the public health sector in China. From 2011 to 2014, the CS rate appeared to decrease in China, especially in metropolitan areas such as Beijing, Shanghai, Hangzhou, and Tianjin [19].

However, the CDMR rate was not included among regulatory monitoring indicators in China, and few studies have examined the CDMR from a policy perspective in China. Previous studies focused on the protective and risk factors of CDMR [20], describing the CDMR rate during a selected period [21], or the outcomes after CDMR [22]. Thus, this study aimed to examine the effectiveness of institutional and policy interventions in reducing the CDMR rate in Wenzhou (a coastal city situated in south-eastern Zhejiang Province), China. The results could provide useful information to other regions in China or other countries that plan to address high CDMR rates.

## Materials and methods

### Ethical approval

This study was approved by the Ethics Committee of Wenzhou Medical University (Code of the ethical approval: 2016–051).

### Data sources

This retrospective pre-/post-intervention study focused on the maternity departments of three tertiary-level public hospitals (labeled Hospital I, Hospital II, and Hospital III in this study) in Wenzhou, China. According to the database of the Wenzhou Health and Family Planning Committee, the number of deliveries in these three hospitals accounted for 31.2% of the total number of deliveries in the Wenzhou Prefecture from 2009 to 2014. The delivery data were collected separately from the three hospitals’ information systems from January 2006 to December 2014, and 131,312 deliveries were included in our study. The dataset contains information such as hospitalization number, mother’s age, date of admission, length of hospital stay, principal diagnosis, and mode of delivery.

### Interventions

#### Institutional interventions

As the main birth delivery hospitals in Wenzhou, these three hospitals developed a similar multifaceted strategy to tackle the high CDMR rate in January 2008. The strategy mainly comprised three aspects. First, the following actions were taken with respect to mothers and their families: (a) Face-to-face health education was provided by doctors, nurses, and nutritionists to mothers and their families once or twice a week in a hospital. The discussions involved the advantages and disadvantages of CS and vaginal delivery, advocating vaginal delivery, nutrition for pregnancy, and breast feeding. Moreover, the contents were also displayed on TV and billboards at hospital outpatient service halls and inpatient wards at the obstetrics departments. (b) When preparing for CDMR, obstetricians shared the potential risks of CS with mothers, and then the mothers were asked to sign a medical informed consent form for CDMR.

Second, the following actions were taken with respect to obstetricians and midwives: (a) They were asked to participate in training programs every year, including for honing necessary skills for problematic child delivery and improving the procedures for emergency obstetric care. (b) Administrators and obstetricians issued specific CS indications and guidelines, which would be adjusted according to developments in the field of obstetrics. Then, the hospitals asked them to perform CS based on these guidelines and emphasize reducing CDMR. Additionally, the professional groups would conduct a monthly audit of whether medically unnecessary CS procedures were performed. Third, the following alternative birth delivery methods were promoted to mothers: (a) painless childbirth through intravertebral anesthesia and (b) one-to-one doula delivery by midwives.

#### Policy interventions

Since 2011, the Chinese central and local governments have developed a series of health policies to decrease the high CS rate by controlling CDMR rate such as in Wenzhou. These policies involved two approaches. One consisted of development plans, including the Regulation for the Management of Maternal Health Care and the Norms of Maternal Health Care [15], the Project of Maternal and Child Health During the 12th Five-Year Plan in Zhejiang Province [16], and the Development Plan for Women in Wenzhou [17]. The other comprised evaluation criteria, including the Medical Quality Management and Control Indicators for Tertiary Comprehensive Hospitals [18]. These policies require providers of maternal and child health care to encourage mothers to choose vaginal delivery, rigorously control indications for CS, and to strictly curb CDMR (Table 1). It is worth noting that the CS rate is included among the Chinese Ministry of Health regulatory indicators for the health sector. Health-care providers will face regulatory or financial penalties if they do not maintain the CS rate within a reasonable range.

**Table 1:** Details of policy interventions for reducing CS and CDMR rates.

Policy	Details of policy intervention
**Development plans**	
The Regulation for the Management of Maternal Health Care and the Norms of Maternal Health Care	a) Encourage mothers to choose vaginal delivery; b) Should strictly control indications for CS; c) Should strictly control CDMR.
The Project of Maternal and Child Health During the 12th Five-Year Plan in Zhejiang Province	Reduce the CS rate in Zhejiang Province.
The Development Plan for Women in Wenzhou	a) Enhance health education about maternal health; b) Popularize knowledge about perinatal health care; c) Reduce the CS rate in Wenzhou area.
**Evaluation criteria**	
Medical Quality Management and Control Indicators for Tertiary Comprehensive Hospitals	The CS rate was included among patient safety indicators.

### Data analysis

CDMR is a cesarean section on maternal request at term, which lacks any medical or obstetric indications [23]. CS with medical indications can be divided into CS with absolute medical indications and CS with relative medical indications [24, 25]. This study defines cesarean delivery without absolute medical indications and relative medical indications as CDMR according to the classification standards above. We distinguished three periods according to the start of institutional and policy interventions: the pre-intervention period (Pre-intervention; 2006–2008), the first post-intervention period (Post-intervention I; 2009–2010, with only institutional interventions), and the second post-intervention period (Post-intervention II; 2011–2014, with institutional and policy interventions). Next, we examined the CS and CDMR rates during the three periods and, finally, used predictive models to calculate the probability of undergoing CDMR during each period.

### Statistical analysis

We examined changes in the CS and CDMR rates in three periods through a chi-square test. Then, a binary logistic analysis was used to identify whether age and different periods have an effect on choosing CDMR. The variable assignments were shown in Table 2.

**Table 2:** Variables and their assignments in the binary logistic regression model.

Variable	Assignment of categories
Y(Get CDMR?)	1 = Yes; 0 = No
X_1_ (Period)	1 = Pre-intervention (2006–2008); 2 = Post-intervention I (2009–2010) 3 = Post-intervention II (2011–2014)
X_2_ (Age)	1 = Mother’s age under 24; 2 = Mother’s age from 24 to 34; 3 = Mother’s age above 34

Finally, a predictive model was constructed to evaluate the effectiveness of these interventions. In the predictive model, P(Y = 1) is the probability that a woman undergoes CDMR; this probability is related to the independent variables X_1_ and X_2_ in the binary logistic analysis model [26]. The predictive model for performing CDMR [27] is

$$\mathrm{Logit}\left(P\right)=\ln\left(\frac{P}{1-P}\right)=\alpha +\beta_{1}X_{1}+\beta_{2}X_{2}$$ (1)

$$P\left(CDMR\right)=\frac{EXP\left(\alpha +\beta_{1}X_{1}+\beta_{2}X_{2}\right)}{1+EXP\left(\alpha +\beta_{1}X_{1}+\beta_{2}X_{2}\right)}$$ (2)

where α refers to the intercept; X_i_ and β_i_ (i = 1,2) refer to the independent variables and their coefficients in the binary logistic regression, respectively; and P(CDMR) captures the probability that a woman undergoes CDMR according to different periods (X_1_) and different age groups (X_2_).

## Results

### Sample characteristics

In total, 131,312 deliveries between 2006 and 2014 were included in our study, out of which 65,247 (49.7%) were vaginal deliveries and 66,065 (50.3%) cesarean deliveries. The median age of mothers was 27 (P_25_–P_75_: 25–31).

### Movement of CS and CDMR rates

**Fig 1:**
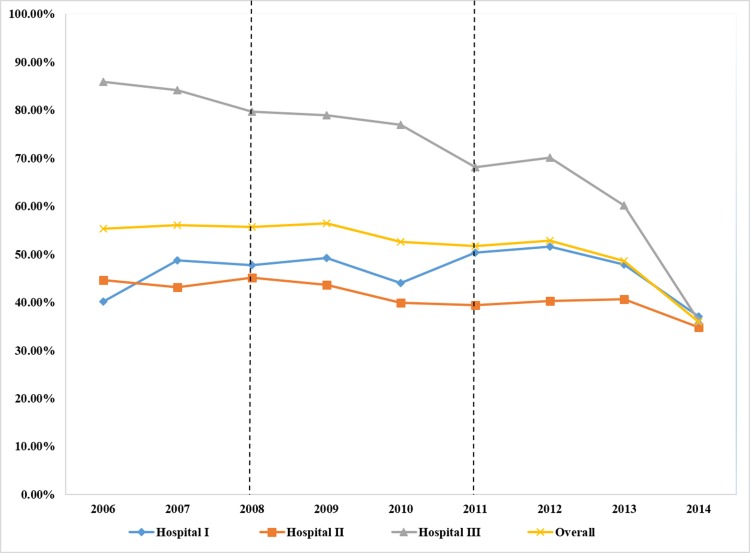
Trends in the CS rate (%) from 2006 to 2014.

**Fig 2:**
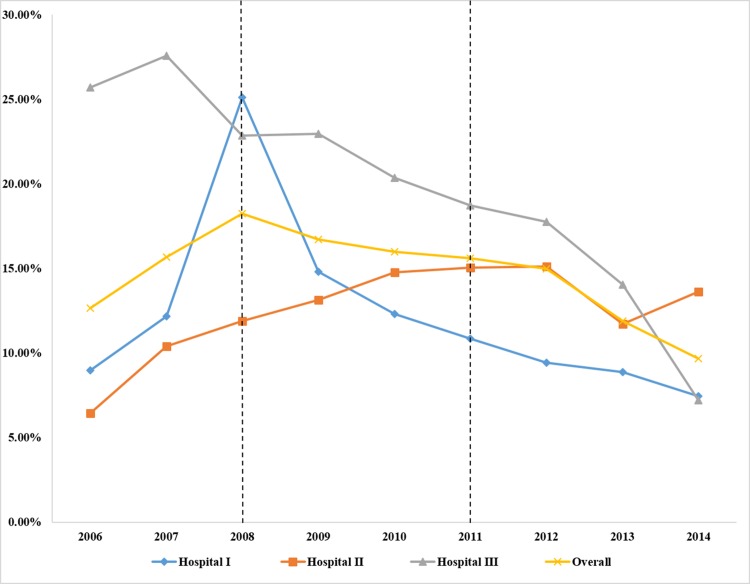
Trends in the CDMR rate (%) from 2006 to 2014.

As shown in Fig 1, the overall CS rate was approximately 55–56% between 2006 and 2008, and declined after 2009. The overall CDMR rate increased rapidly, from 12.65% in 2006 to 18.25% in 2008, and then decreased quickly, from 18.25% in 2008 to 9.66% in 2014 (Fig 2).

### Evaluating the effect of interventions

After institutional interventions, the overall CS rate declined by 1.29% (p = 0.002) between 2006–2008 and 2009–2010 (Table 3). At the same time, the average annual growth rate (AAGR) of the CS rate decreased from 0.29% to -6.73%. A non-significant increase in the CDMR rate was observed during Pre-intervention (15.76%) and Post-intervention I (16.34%; p = 0.053), but the AAGR of the CDMR rate declined rapidly from 20.11% to -4.30%.

**Table 3:** Effect of institutional and policy interventions on the CS and CDMR rates.

	Pre-intervention	Post-intervention		*χ*^*2*^	*p-value*
	2006–2008	2009–2010	2011–2014		
Hospital I					
Total sample size (%)	8,601 (100.00)	5,795 (100.00)	14,856 (100.00)		
CS cases (%)	3,940 (45.81)	2,692 (46.45)	6,689 (45.03)	3.79	> 0.05
Medical CS cases (%)	2,573 (29.92)	1,910 (32.96)	5,380 (36.21)	90.57	< 0.01
CDMR cases (%)	1,367 (15.89)	782 (13.49)	1,309 (8.81)	281.51	< 0.01
Hospital II					
Total sample size (%)	15,948 (100.00)	12,063 (100.00)	30,452 (100.00)		
CS cases (%)	7,065 (44.30)	5,033 (41.72)	11,781 (38.69)	141.33	< 0.01
Medical CS cases (%)	5,494 (34.45)	3,346 (27.74)	7,570 (24.86)	477.65	< 0.01
CDMR cases (%)	1,571 (9.85)	1,687 (13.98)	4,211 (13.83)	168.546	< 0.01
Hospital III					
Total sample size (%)	9,780 (100.00)	8,483 (100.00)	25,334 (100.00)		
CS cases (%)	8,119 (83.02)	6,611 (77.93)	14,135 (55.79)	2984.13	< 0.01
Medical CS cases (%)	5,647 (57.74)	4,776 (56.30)	10,716 (42.30)	931.03	< 0.01
CDMR cases (%)	2,472 (25.28)	1,835 (21.63)	3,419 (13.50)	782.01	< 0.01
Overall					
Total sample size (%)	34,329 (100.00)	26,341 (100.00)	70,642 (100.00)		
CS cases (%)	19,124 (55.71)	14,336 (54.42)	32,605 (46.16)	1066.31	< 0.01
Medical CS cases (%)	13,714 (39.95)	10,032 (38.09)	23,666 (33.50)	472.20	< 0.01
CDMR cases (%)	5,410 (15.76)	4,304 (16.34)	8,939 (12.65)	305.97	< 0.01

After policy interventions, the overall CS rate decreased significantly from 54.42% to 46.16% (p < 0.001), as shown in Table 3. The AAGR of the CS rate in the 2011–2014 period was lower than in the 2009–2010 period. The overall CDMR rate declined by 3.69% (p < 0.001), and the AAGR of the CDMR rate decreased from -4.30% to -14.77% after policy interventions were applied.

### Probability of performing CDMR

First, results of the binary logistic regression analyses were presented in Table 4.

**Table 4:** Binary logistic regression results using delivery data.

Variables		*β*	*OR(95%CI)*	*p-value*
Hospital I				
	Period	-0.36	0.70(0.67~0.73)	< 0.01
	Age	0.28	1.32(1.24~1.41)	< 0.01
	Constant	-1.8	0.17	< 0.01
Hospital II				
	Period	0.15	1.16(1.13~1.20)	< 0.01
	Age	0.47	1.60(1.50~1.69)	< 0.01
	Constant	-3.18	0.04	< 0.01
Hospital III				
	Period	-0.39	0.68(0.66~0.70)	< 0.01
	Age	-0.25	0.78(0.74~0.82)	< 0.01
	Constant	-0.15	0.86	< 0.01
Overall				
	Period	-0.15	0.86(0.85~0.88)	< 0.01
	Age	0.17	1.19(1.15~1.23)	< 0.01
	Constant	-1.81	0.16	< 0.01

Next, to construct the predictive model for performing CDMR, two statistically significant independent variables (Period and Age) with their estimated coefficients were added to Eq (2). The predictive model is as follows.

$$\mathrm{For}\;\mathrm{Hospital}\;I,\;P\left(CDMR\right)=\frac{EXP\left(-1.80-0{.36X}_{1}+0.28X_{2}\right)}{1+EXP\left(-1.80-0{.36X}_{1}+0.28X_{2}\right)}$$

$$\mathrm{For}\;\mathrm{Hospital}\;\mathrm{II},\;P\left(CDMR\right)=\frac{EXP\left(-3.18+0{.15X}_{1}+0.47X_{2}\right)}{1+EXP\left(-3.18+0{.15X}_{1}+0.47X_{2}\right)}$$

$$\mathrm{For}\;\mathrm{Hospital}\;\mathrm{III},\;P\left(CDMR\right)=\frac{EXP\left(-0.15-0{.39X}_{1}-0.25X_{2}\right)}{1+EXP\left(-0.15-0{.39X}_{1}-0.25X_{2}\right)}$$

$$\mathrm{Overall},\;P\left(CDMR\right)=\frac{EXP\left(-1.81-0{.15X}_{1}+0.17X_{2}\right)}{1+EXP\left(-1.81-0{.15X}_{1}+0.17X_{2}\right)}$$

The results from employing the predictive models are shown in Table 5; we observe that the overall probability of performing CDMR decreased in all three age groups after institutional interventions (2010–2011). After policy interventions were applied during 2011–2014, the overall probability declined. For example, the overall probability of opting for CDMR in the 24–34 age group was 0.17, 0.15, and 0.13 in the Pre-intervention, Post-intervention I, and Post-intervention II, respectively.

**Table 5:** Probability (%) of performing CDMR pre- and post-intervention.

Period		Mother’s age		
		< 24	24–34	> 34
Hospital I				
	2006–2008	0.13	0.17	0.21
	2009–2010	0.10	0.12	0.16
	2011–2014	0.07	0.09	0.12
Hospital II				
	2006–2008	0.07	0.11	0.17
	2009–2010	0.08	0.13	0.19
	2011–2014	0.09	0.14	0.21
Hospital III				
	2006–2008	0.31	0.26	0.22
	2009–2010	0.24	0.19	0.16
	2011–2014	0.17	0.14	0.11
Overall				
	2006–2008	0.14	0.17	0.19
	2009–2010	0.13	0.15	0.17
	2011–2014	0.11	0.13	0.15

## Discussion and conclusions

Our study shows that after institutional and policy interventions, the overall CDMR rate decreased. Additionally, the overall probability of mothers undergoing CDMR in the Post-intervention I and II periods was lower than in the Pre-intervention period.

The results show that institutional interventions can reduce the CDMR rate, a finding consistent with previous studies [28–30]. Potential reasons are: First, many mothers think that CS is safe [31], but health education and the requirement to sign a medical informed consent form for CDMR change this stereotypical attitude of mothers and their families towards CS, especially towards CDMR. Second, training programs and guidelines with medical and obstetric indications enhanced physicians’ ability to manage vaginal labor and optimized the process of delivery care. Last, fear of childbirth was an important reason for mothers to choose CDMR[32, 33]. Intravertebral anesthesia can reduce mothers’ pain during vaginal delivery. A doula is a supportive mothers’ companion who can share the experience of labor and encourage mothers to more readily face vaginal delivery [34, 35].

Further, this study revealed that policy interventions can decrease the CDMR rate, and this finding may be attributed to the fact that the CS rate is required to be included in hospital assessment. To reduce the CS and CDMR rates, other countries focused on financial incentives [36, 37]. In China today, local governments continue to have a direct financial and management role in public hospitals, aiding policy interventions in achieving the ideal effect.

It is remarkable that institutional and policy interventions for Hospital I were effective in reducing the CDMR rate but not the CS rate. This finding may be explained by the fact that Hospital I is the critical care medical center of Southern Zhejiang, and therefore, it must receive patients with more critical conditions. Mothers with critical conditions will more likely seek health care at Hospital I. Therefore, Hospital I had a relatively greater share of CS deliveries.

However, it is worth noting that institutional and policy interventions had no effect on reducing the CDMR rate in Hospital II, inconsistent with the findings for Hospitals I and III. The additional fees for CDMR could be responsible for this finding. Unlike in Hospitals I and III, an additional charge of ¥800 (approximately $129.24) is imposed for selecting delivery time in Hospital II. Therefore, this additional charge increased the potential risk for supplier-induced demand, which in turn reduced the effectiveness of institutional and policy interventions, even when the CS rate decreased after these interventions [38, 39].

In summary, our study provides evidence that institutional and policy interventions could reduce the high CDMR rate in China. Given that the CS rate was simpler and more convenient to monitor, it was included among the assessment indicators for public hospitals in most policies, whereas the CDMR rate was not. This situation created an obstacle to effectively control CDMR. To control CDMR more effectively, it should be included in the assessment mechanism in further policymaking. What is more, supplier-induced demand can also reduce the effectiveness of these interventions. Thus, more research is needed to explore the potential supplier-induced demand for CDMR in similar hospitals. In short, popularizing institutional interventions, strengthening the performance assessment mechanism for public hospitals, and making full use of the policy to reduce CDMR at the national level are of paramount importance for other countries facing similar circumstances.

The universal two-child policy in today’s China has created an opportunity to address the high CDMR rate, especially in the case of primiparas undergoing CDMR. Thus, the health sector and administrative departments in charge of health care should seize this opportunity to reduce the CDMR rate and carry out a series of studies on CS interventions under the universal two-child policy.

## Innovation and limitations

This study used 9-year delivery data collected from three hospitals’ information systems to allow observation of changes in CDMR before and after institutional and policy interventions over a long period. Additionally, the predictive model for performing CDMR could reveal the effectiveness of these interventions accurately and comprehensively.

Our study has some limitations. First, although these three tertiary-level public hospitals are the main birth hospitals in Wenzhou and can represent the effectiveness of institutional and policy interventions in tertiary-level hospitals, primary, secondary, and private hospitals were not included in this study. Therefore, further research is needed to evaluate the effectiveness of institutional and policy interventions in reducing the CDMR rate in other types of hospitals, such as primary, secondary, and private hospitals. Second, our study focused on the Wenzhou area, so further studies using a similar analysis should be carried out in other regions in China, such as Hangzhou, Beijing, and Shanghai, to evaluate the effect of these interventions. This would help generalize our research findings on these interventions in China to other countries facing similar circumstances. Third, other studies revealed a substitution effect between the assisted vaginal delivery rate and the CS rate [40]. Our study focused only on CS and CDMR rates owing to the limited information we were able to collect from the hospitals’ information systems. Therefore, future studies need to evaluate the impact of institutional and policy changes on the assisted vaginal delivery rate. What is more, the number of times a woman has given birth can influence her decision to choose CDMR [41]. We only concentrated on the overall CDMR rate because of the limitation of our delivery data; thus, future research studies should compare the CDMR rate of a woman in her first pregnancy with the corresponding rate of women waiting for their second baby. Last, the data collected from the hospitals’ information systems were insufficient for us to determine which intervention was most effective. Thus, we focused only on the effect of comprehensive interventions. Future studies could evaluate the effectiveness of different interventions to reduce the CDMR rate.

## Abbreviations

CS: Cesarean section
CDMR: Cesarean delivery on maternal request
AAGR: Average annual growth rate
